# Climate change is the primary driver of white‐tailed deer (*Odocoileus virginianus*) range expansion at the northern extent of its range; land use is secondary

**DOI:** 10.1002/ece3.2316

**Published:** 2016-08-18

**Authors:** Kimberly L. Dawe, Stan Boutin

**Affiliations:** ^1^ Quest University3200 University BoulevardSquamishBCCanadaV8B 0N8; ^2^ Department of Biological SciencesUniversity of AlbertaCW405 Biological Sciences BuildingEdmontonABCanadaT6G 2E9

**Keywords:** Climate change, development, distribution, range limit, species distribution model, winter severity

## Abstract

Quantifying the relative influence of multiple mechanisms driving recent range expansion of non‐native species is essential for predicting future changes and for informing adaptation and management plans to protect native species. White‐tailed deer (*Odocoileus virginianus*) have been expanding their range into the North American boreal forest over the last half of the *20th* century. This has already altered predator–prey dynamics in Alberta, Canada, where the distribution likely reaches the northern extent of its continuous range. Although current white‐tailed deer distribution is explained by both climate and human land use, the influence each factor had on the observed range expansion would depend on the spatial and temporal pattern of these changes. Our objective was to quantify the relative importance of land use and climate change as drivers of white‐tailed deer range expansion and to predict decadal changes in white‐tailed deer distribution in northern Alberta for the first half of the *21st* century. An existing species distribution model was used to predict past decadal distributions of white‐tailed deer which were validated using independent data. The effects of climate and land use change were isolated by comparing predictions under theoretical “*no‐change* between decades” scenarios, for each factor, to predictions under observed climate and land use change. Climate changes led to more than 88%, by area, of the increases in probability of white‐tailed deer presence across all decades. The distribution is predicted to extend 100 km further north across the northeastern Alberta boreal forest as climate continues to change over the first half of the 21st century.

## Introduction

Understanding the mechanisms leading to changes in species' ranges is a fundamental question in ecology. Parmesan and Yohe (2003) recorded an average poleward range expansion of 6.1 km per decade across 99 species in response to 20th‐*century* climate change. However, 19% of changes at the poleward and upper elevational range boundaries studied shifted in a direction that was inconsistent with changing climate (Parmesan and Yohe 2003). Hockey et al. (2011) found that 12.8% of South African bird species that expanded their ranges shifted in a direction consistent with climate change, while 13.1% shifted in a direction more consistent with land use change. Much emphasis has been on the impact of climate on range boundaries (examples include: Peterson et al. 2002; Parra and Monahan 2008); however, changes in landscapes as a result of human land use could be an equally or more important driver of species range expansion. To date, few studies have attempted to quantify the relative impact of multiple large‐scale stressors on species range expansion (but see Melles et al. 2011; Rubidge et al. 2011; Struebig et al. 2015).

White‐tailed deer (*Odocoileus virginianus*) have been expanding their range into the North American boreal forest over the last half of the *20th* century (Webb 1967; Veitch 2001). At the start of the 20th century, the northern range edge for white‐tailed deer roughly followed the southern edge of the boreal forest from Alberta to New Brunswick, Canada (McCabe and McCabe 1984). The range edge has moved north across the Canadian distribution; however, changes in Alberta have been particularly extensive. By 1960, white‐tailed deer occurred in agricultural areas in northwestern Alberta and along the Athabasca River running between Athabasca and Fort McMurray (Webb 1967), and between 1969 and 2001, they increased in occurrence in northeastern Alberta (Charest 2005). Between the 1990s and 2000s, there was a 17.5‐fold increase in white‐tailed deer abundance in NE Alberta (Latham et al. 2011). The northern extent of sightings now extends as far north as Norman Wells, North West Territories, and Dawson, Yukon (65.17° and 64.04° latitude, respectively), and presence is common throughout southwestern Northwest Territories and northeastern BC (T. Jung, personal communication, 12 June 2014, Veitch 2001).

The negative consequences of species range shifts and our ability to mitigate or manage those consequences have been less considered than the ability of species to track changes in factors driving range limits. The population increase and expansion of white‐tailed deer has already led to increased gr*ay* wolf (*Canis lupus*) abundance and elevated predation on threatened woodland caribou (*Rangifer tarandus caribou*; Dale et al. 1994; COSEWIC 2002, Latham et al. 2011). The consequences of white‐tailed deer range expansion may further include increases in invasibility of ecosystems to exotic plant species (Parker et al. 2006), increased human–wildlife conflict (White and Ward 2010), and increased disease spread (Bar‐David et al. 2006; Oyer et al. 2007). Where overabundant, deer herbivory also affects nutrient cycles (Bardgett and Wardle 2003), vegetation community structure (Potvin et al. 2003, Côté et al. 2004), and successional pathways (Hobbs 1996). Quantifying the relative influence of multiple mechanisms driving recent range expansion is essential for predicting future changes and for informing adaptation and management plans to protect native species.

Dawe et al. (2014) identified that climate, particularly an index of winter severity, was the most important individual factor determining current white‐tailed deer distribution in boreal Alberta. Human land use (as measured by total land use footprint) also acted to substantially increase the probability of white‐tailed deer presence (Dawe et al. 2014). There have been concurrent changes in climate and land use in the region and either climate or land use, or both may have facilitated spread of white‐tailed deer. The influence each factor had on the observed range expansion of white‐tailed deer would depend on the spatial and temporal pattern of these changes in the past. Similarly, the future trajectory of white‐tailed deer expansion could differ widely depending on whether climate or land use drives expansion. Further changes in climate are expected to occur in the region by the end of the *21st* century (Christensen et al. 2007). Temperature increases may exceed 5°C and precipitation is also expected to increase, although the proportion of precipitation falling as snow, the length of the snow cover period and/or the depth of the snow pack may decrease (Hayhoe et al. 2004; Christensen et al. 2007). In contrast, land use changes are not expected to continue as they have in the past. Agriculture has reached its northern limit in Canada and is expected to expand only east and west from existing margins (Hamley 1992). Forestry sector cutting will occur within already established forest management areas and energy sector developments are projected to intensify within the oil sands regions, however, likely will not change much in a northerly direction (National Energy Review Board 2006).

Our objective was to quantify the relative importance of land use and climate change as drivers of white‐tailed deer range expansion and to predict decadal changes in white‐tailed deer distribution in northern Alberta for the first half of the *21st* century. We used the white‐tailed deer species distribution model (SDM) from Dawe (2011) to predict past decadal distributions of white‐tailed deer in northern Alberta and validated those predictions in each historic decade using independent data. Because both climate and land use variables may have changed simultaneously, we elucidate which of these factors changed the most, relative to their influence on predicted presence of white‐tailed deer. Under a climate change hypothesis, we predict that changes in climate variables will explain the spatial and temporal changes in the probability of white‐tailed deer presence over time. Alternatively, under a land use hypothesis, temporal changes in the probability of white‐tailed deer presence will be the result of new forestry, oil and gas, and/or agriculture footprint, while the spatial pattern of spread will follow closely to the change in land use footprint. We isolated the effects of climate and land use change by comparing predictions under theoretical “no‐change” scenarios for each mechanism between decades to predictions under observed climate and land use change. Finally, based on these results, we predicted future distributions of white‐tailed deer in the western boreal forest.

## Methods

The overall workflow for this analysis involved: (1) selecting an appropriate model to describe white‐tailed deer distribution; (2) *a*cquiring data to population the model covariates through past decades; (3) predicting the presence of deer through that time period (hindcasting); and (4) testing the model predictions using independent data and standard validation metrics. The relative influence of climate change and land use factors *was* then investigated, using the time validated model and temporal manipulation of model covariates. Finally, covariates were extrapolated to facilitate future distribution predictions.

### Study region

**Figure 1:**
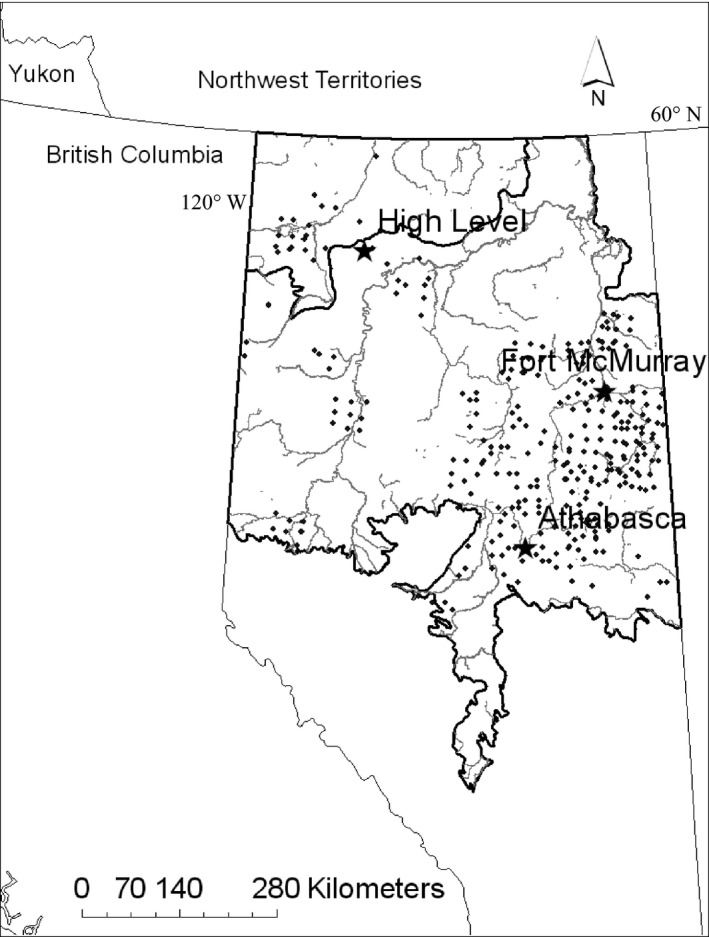
Study area. Dark black outline indicates the two ecozone boundaries in the study area. The light gray lines show major rivers. Black dots are sampling locations for the training data. Stars show the location of towns mentioned in the text.

The study region covers the Alberta boreal forest natural region within the boreal and taiga plains ecozones of Alberta, approximately 380,000 km^2^ (Fig. 1). The region is a mosaic of upland forests composed of white spruce (*Picea glauca*), jack pine (*Pinus banksiana*), trembling aspen (*Populus tremuloides*), and balsam poplar (*Populus balsamifera*), and peatland complexes composed of shrub and fen wetlands, and black spruce (*Picea mariana*) and tamarack (*Larix larcina*) bogs. Mean temperatures from the warmest and coldest month are 15.7 and −19.2°C, respectively, and mean annual precipitation is 469 mm (Downing and Pettepiece 2006). Both temperature and precipitation have changed in northwestern North America since 1908 (Jarvis and Linder 2000; Stone et al. 2002; Nemani et al. 2003; Lemke et al. 2007; Strong et al. 2009). Snow melt is occurring 8 days earlier in northern Alaska compared to pre‐1960 periods due to decreased snow fall and warmer springs, a trend that has likely occurred across northwestern North America (Stone et al. 2002; Lemke et al. 2007). Concurrent changes in the length of the growing season have also led to earlier plant growth in North America and higher productivity (Jarvis and Linder 2000; Nemani et al. 2003). Northern Alberta has also undergone rapid industrial development in the last half of the *20th* century. Agriculture quickly extended through northwestern Alberta reaching 58.31° latitude by the late 1950s, which is the most northern agricultural development in North America (Hamley 1992). Forest harvesting more than tripled in Alberta from 1984 to 2000, with harvest going from 6.5 to 21.9 million m^3^ (Smith et al. 2003). Oil and gas discoveries were made in the province in the late 1940s, with large production increases occurring in the 1970s and early 1990s. The number of well pads in the province increased from approximately 2000 in 1975 to almost 160,000 in 2000 (Schneider 2002). However, there is considerable spatial variation in climate parameters and in land use footprint across the extent of the study area (Dawe et al. 2014).

### Species distribution model

The white‐tailed deer SDM was a generalized linear model with binomial family and logit link containing variables for winter severity, length of the growing season, land cover type, and total nonlinear land use footprint (i.e., linear features such as roads, pipelines, and seismic lines were not included; Dawe et al. 2014). The model was selected from a suite of a priori climate, land cover, land use, and spatial models, according to the lowest AIC score and highest weight of evidence (*w*_*i*_ = 1.0) (Anderson 2008). There was no autocorrelation in the residuals of the selected model, suggesting there were no spatial processes unaccounted for in the model (Dawe et al. 2014). The response data for the SDM were presence and absence data from 299 snow‐tracking sample sites, collected between 2002 and 2009 in the boreal and taiga plains of Northern Alberta (Fig. 1). Sample sites were selected according to a stratified random or systematic design, and snow track sampling was conducted by the Alberta Biodiversity Monitoring Institute (*N* = 127), the Integrated Landscape Management *laboratory* at the University of Alberta (*N* = 151) or a combined effort between these groups (*N* = 21). Two methods were used to collect the data: a *9*‐km triangular‐shaped transect sampled on foot, and a 10‐km linear transect sampled on snowmobile (Dawe et al. 2014). While Dawe et al. (2014) reported a generalized version of the model, Dawe (2011) produced a model accounting for sample method. The model for the transect method was applied here (Dawe 2011).

Winter severity in the model was based on an index by DelGiudice et al. (2002) that sums the number of days between November and April that have a minimum temperature below −17.7°C and/or snow depth above 38 cm. Dawe and Boutin 2012 adapted the index for use with snow water equivalent data (WSIswe). The index was calculated annually using temperature and precipitation data interpolated from climate stations using thin‐plate smoothing splines, obtained from Natural Resources Canada (Hutchinson et al. 2009). These data were at a spatial grain of 10 km. Growing season started when the mean daily temperature was equal or greater than 5°C for at least five consecutive days, beginning on March 1, and ended when the minimum temperature reached −2°C after 1 August. Natural Resources Canada developed this metric using the same temperature data used for calculation of the WSIswe, but at an 8.36‐km grain size (Hutchinson et al. 2009). In the model, WSIswe and growing season were averaged over the last half of the *20th* century according to data availability (WSIswe: 1961–2002 and growing season length: 1950–1999). Long‐term averaged climate variables have been found to have higher predictive power than shorter term climate variables in ecological models and models containing long‐term averaged WSIswe variables *out*competed models containing WSIswe from the 2 years prior to sampling (Hallett et al. 2004; Melles et al. 2011; Dawe et al. 2014). Total land use footprint was taken as the cumulative proportion of agriculture, forestry cut blocks, and well pads within a 5*00*‐m region around the sampling unit, and land cover variables were the proportion of deciduous forest and wetland land cover types, respectively, within that 500 m buffer.

### Hindcasting

To predict past distributions, decadal landscapes were established for each of the SDM variables. Using GIS analysis, a 10 × 1 km east–west grid was placed across the boreal and taiga plains ecozones of northern Alberta. This is the same scale as transect data used to develop the SDM. The SDM equation was calculated for all grid squares and converted to probabilities using the inverse logit:

$$\text{Probability of deer presence}=\frac{\exp^{\mathrm{model}}}{1+\exp^{\mathrm{model}}}$$

where exp^model^ is the exponent of the model:

$$\begin{array}{cc} & \text{Logit Probability of deer presence}\\ & \;=-10.12+4.98\times \text{Proportion Deciduous}-3.11\\ & \;\times \text{Proportion wetland}+9.79\\ & \;\times \text{Proportion land use}-0.07\\ & \;\times \text{WSIswe}+0.11\times \text{growing season}. \end{array}$$

#### Environmental variables

Climate variables were calculated for the center of each grid cell. The mean WSIswe from 1961 to the beginning of the decade of interest and the mean growing season length from 1950 to the beginning of each decade of interest were included in models for hind‐casted predictions.

The proportion of each land use footprint was calculated for each grid cell, and these proportions were added to give the total footprint. We accounted for areas where land uses overlapped to ensure total land use proportion could not exceed one. Except for the agriculture data, described below, the data used to describe hind‐casted landscapes were the same as those used to develop the SDM (Dawe 2011).

Digital data on well pads were obtained from the Alberta Base Features data set from 2008, which is the digital representation of the 1:20,000 maps for the region. Well‐pad point locations were expanded to 100 m^2^ to account for the size of forest clearing associated with well establishment (Hird et al. 2009). Well‐pad development date was available for more than 99% of the wells on the landscape, otherwise license date was available. The well‐pad data covered all well pads from 1900 to 2010. There is no regulation requiring reforestation on abandoned well sites, and natural regeneration is hindered by these disturbances (Osko and MacFarlane 2001; Schneider 2002). For this reason, we used the cumulative total of wells for subsequent decades.

Cut‐block data were obtained from Alberta Biodiversity Monitoring Institute in the form of Alberta Vegetation Inventory data, based on 1987–2009 1:20,000 scale aerial photographs, with some field verification (Nesby 1997). Thirty percent of the cut blocks in the data set were missing year of cut information. Decadal model calculations were completed with these cut blocks included and excluded to investigate the impact of this on inferences. Cut blocks developed prior to 1970 were not included in the 2000s footprint because after approximately 30 years, browse on cut blocks is expected to have grown too high to be available for deer.

We used agricultural ecumene data for 1991 and 2006 from Statistics Canada (Werschler 1995) to represent the change in agriculture through time. These data summarize the agricultural landscape according to areas with high levels of agricultural products produced for sale. To be included in the spatial representation of agriculture, census coverage areas are ranked according to the aerial ratio of developed to undeveloped land and added to the ecumene from highest to lowest ranking until the total area of improved and unimproved land for the region is reached (Werschler 1995). This removes small isolated farms from the ecumene (Werschler 1995). The ecumene data align spatial boundaries with other geographic features rather than mapping boundaries by GPS coordinates, so may have inaccuracies in spatial boundaries (Statistics Canada 2006). We removed any regions from the ecumene data that fell outside the Alberta white zone boundary, which delineates the region available for agricultural development. We also assumed that agricultural footprint from 1991 would remain on the landscape in 2006, even if not under production, so regions developed in 1991 were included in the total agriculture footprint for the 2000 decade. We used the 1991 agriculture ecumene to describe the 1970s and 1980s agricultural footprint, and the adjusted 2006 ecumene data to describe the 1990s and 2000s agricultural footprint (Werschler 1995; Statistics Canada 2006). There was only a 4% increase in the area of occupied farmland between 1971 and 1991 in Alberta (Statistics Canada 2007; Stelfox accessed 2011) and the change in agricultural area between the 1991 and 2006 ecumene only accounts for 4% of the study region. Although the coarse temporal resolution of the agricultural data may reduce predictive accuracy in the regions that experienced agricultural expansion, this uncertainty applies to a small proportion of the study area, and only within close spatial proximity to the known agricultural boundaries, so should not affect overall inferences.

Data from Alberta Ground Cover Characterization (Sanchez‐Azofeifa et al. 2004) were used to calculate the proportion of deciduous forest and wetlands for the training and validation data sets. Deciduous forests were defined as greater than 80% occurrence of deciduous species. The Alberta Ground Cover Characterization data were developed using Landsat Thematic Mapper and Landsat 7 Enhanced Thematic Mapper satellite imagery with images acquired in 1999–2002 (Sanchez‐Azofeifa et al. 2004). Land use developments on the landscape prior to 1999 would have been included in the layer as urban and industrial, agriculture, clear‐cuts, or vegetation classification according to regrowth at the location.

Differences in spatial and temporal resolution of GIS layers led to total proportions of land use and land cover that exceeded one in some cases. In this case, the total proportion of land use and land cover in a grid cell was reduced to one by reducing the land cover variables. The land cover variables were given lower priority because the data were at coarser resolution than the land use data [resolution of variables is described in further detail in Dawe et al. (2014)]. The amount by which land cover was reduced was subtracted from the proportion of deciduous and wetland land cover proportional to their coverage in the grid cell. In this way, land use development in any decade functionally erased land cover types from the landscape.

#### Independent data

**Figure 2:**
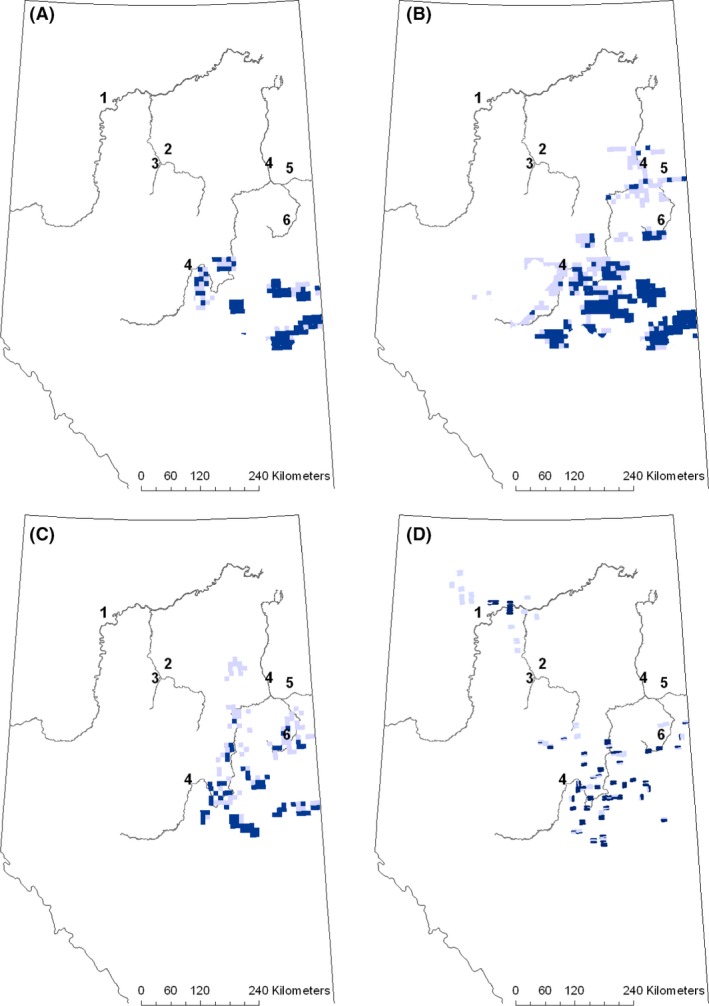
Validation data sets for the 1970s (A), 1980s (B), 1990s (C), and 2000s (D). Dark cells indicate presence of white‐tailed deer and light cells indicate absence. The numbers correspond to the Peace (1), Wabasca (2), Loon (3), Athabasca (4), Clearwater (5), and Christina (6) rivers.

Validation is a “vital” component of model building, and validation with independent data is essential to properly evaluating the predictive performance of SDMs (Pearce and Ferrier 2000; Araújo et al. 2005; Guisan and Thuiller 2005; Kharouba et al. 2008). We tested the predictions of the SDM using two independent validation data sets. The first validation data set was aerial survey data, collected in two regions of Alberta: High Level, 58°31′0″ North, 117°8′0″ West and Athabasca, 54°43′0″ North, 113°16′0″ West, and flown using fixed wing aircraft between December 2007 and January 2008 (Fig. 2). A total of 23 randomly selected agriculture sites (5 in High Level and 18 in Athabasca), and 49 forested sites (13 and 36 in High Level and Athabasca, respectively), stratified by high and low forestry footprint, were sampled. Land cover was standardized using Alberta Vegetation Inventory data in a GIS such that all sites had >39% total upland land cover with deciduous forest or deciduous dominated mixed wood covering between 30 and 60% of the site. Three transects, buffered by 300 m to cover the approximate area that could be surveyed from the aircraft, were flown in each 10 km by 10 km site. For each site, we compared the average predicted probability of white‐tailed deer presence in the 2000s from the model to observed presence or absence.

The second validation data set consisted of aerial ungulate survey data from 76 rotary aircraft surveys conducted by Alberta Sustainable Resource Development between 1971 and 1997 (Charest 2005). Survey design varied among surveys, consisting of either random or directed paths to target expected concentrations of white‐tailed deer, transect sampling, full coverage surveys of polygons stratified by land cover, or full coverage surveys stratified by expected deer density. The Western Canada land description system divides Alberta into a grid of 10 km by 10 km townships (McKercher and Wolfe 1986). Survey results were summarized by townships such that a sampled township was scored with a presence if any portion of that township had a presence recorded; otherwise, it was scored as an absence. Once a township was scored with a presence, absences from subsequent surveys in the same township were considered false absences and the township remained as a presence for the analysis. We formed three decadal data sets to test model predictions in the 1970s, 1980s, and 1990s (Fig. 2). For each township, we compared the average predicted probability of white‐tailed deer presence from the model to observed presence or absence.

The validation data are not suitable to model building for the purposes of distinguishing the effects of climate change and land use because they have a limited spatial extent within any given decade, variation in sample design across data sets, and likely critical issues with imperfect detection as a result of the aerial survey collection methods. Variation in sample design among validation data sets is not expected to affect validation statistics. While the survey design will impact the ability to interpret white‐tailed deer presence and abundance within that landscape, the validation data are used only to score presences and absences. Thus, only variation in the ability of different sampling designs to reliably score presence and absence will affect validation statistics. The spatial scale of the validation data sets is larger than that of the model predictions. The validation process tests how well the model predicts independent observations. If the differences in spatial scale between the validation and model data are of concern, it is expected to be reflected in poor validation statistics.

#### Model validation

Three methods of model validation were employed. Predictions from logistic regression models are on a probability scale from 0 to 1, while validation data contain observed binary states of presence or absence. To determine whether model predictions agree with these binary states, a probability threshold must be set to convert predictions to presence or absence. The prevalence in the training data (i.e., the data used to build the original model), which is the number of occurrences divided by the number of observations, is directly related to the model probabilities in a logistic regression model (Liu et al. 2005). It has been suggested as one of the best thresholds for converting probabilities of occurrence to presence/absence data (Liu et al. 2005). We used prevalence in the training data as the threshold for calculating model sensitivity (the proportion of correctly predicted presences) and specificity (the proportion of correctly predicted absences) to investigate trade‐offs in prediction error (Pearce and Ferrier 2000). We also calculated the true skills statistic (TSS), which is the sensitivity + specificity − 1 (Allouche et al. 2006). This statistic has been shown to be more robust to changes in prevalence than the commonly used Cohen's kappa and is gaining use for testing SDM predictions (Allouche et al. 2006; Rubidge et al. 2011). TSS values range from −1 to +1, with a 0 meaning prediction is no better than random, and a value above 0.5 considered to show high predictive power (Allouche et al. 2006; Rubidge et al. 2011).

The area under the receiver operator curve (ROC) is currently the most widely used metric to test the predictions of SDMs because it summarizes performance over a range of threshold values (Lobo et al. 2010). The area under the curve (AUC) measures the model's ability to estimate a higher probability of occurrence for a randomly selected site with the species present compared to a randomly selected site with the species absent; as such, it tests the discrimination ability of a model (Fielding and Bell 1997). The discrimination ability of models producing AUC values between 0.5 and 0.7 is considered poor, between 0.7 and 0.9 is considered good, and above 0.9 is considered very good (Pearce and Ferrier 2000). The ROCR package in R 2.11.1 was used to calculate AUC.

Another component of predictive ability is calibration. The model probabilities indicate the proportion of sites predicted to have that probability value that should be occupied across the region (Pearce and Ferrier 2000). For example, 20% of all grid cells with a predicted probability of 0.20 should be occupied. While a well‐calibrated model will usually have good discriminatory power, the opposite is not true (Pearce and Ferrier 2000). Model calibration can only be tested against validation data collected under a representative sample of the landscape (i.e., random, stratified random etc); thus, model calibration was only tested using the 2000s validation data set. We ran a generalized linear model with a binomial family and logit link using observed presences and absences from the 2000 validation data as the dependent variable, and the associated predicted probabilities as the independent variable. The fitted values from this model were plotted against the predicted probabilities at those sites. Perfect calibration would result in a plotted line with slope 1 and intercept 0. Points falling above or below this line indicate the model under‐ or overestimates the proportion of occupied sites, respectively (Pearce and Ferrier 2000).

#### Spatially and temporally explicit change

To determine where land use was responsible for a change in probability between the 1970 and 1980 decades, we calculated the predicted probabilities in the 1980s for each grid cell, using the land use footprint data from the 1970s and the climate data from the 1980s. The new probability grids, which assume no change in land use between 1970 and 1980 (i.e., changes in the 1980s were not included), were then subtracted from the correctly calculated 1980s probabilities. Conversely, to determine where climate was responsible for a change in probability between the 1970 and 1980 decades, we calculated the predicted probabilities for the 1980s using the land use footprint from 1980 and the climate data from the 1970s. The new probability grids, which assume no change in climate between 1970 and 1980 (i.e., changes in the 1980s were not included), were subtracted from the correctly calculated 1980s probabilities. Where subtracted values equaled zero in both cases, there was no change in probability between decades. Where the absolute value of the difference in probabilities was greatest for climate, climate was the driver of change for that grid cell, and where the absolute value of the difference in probabilities was greatest for land use, land use was the driver of change for that grid cell. This series of calculations was repeated for the changes between 1980 and 1990, and 1990 and 2000. We also identified where changes resulted in the predicted probability of presence crossing the prevalence threshold, theoretically, signifying a change from absence to presence.

### Forecasting

Projections of climate change reported by the Intergovernmental Panel on Climate Change (IPCC) are based on general climate models (GCMs) developed at grid cell sizes ranging from 125 to 400 km and over monthly time periods (Christensen et al. 2007). These GCM projections are not at the spatial or temporal resolution needed for climate variables in the white‐tailed deer SDM. Projections from GCMs under emissions scenarios developed in the *4th* assessment report (the B1 scenario, in particular) and a range of likely outcomes developed in the *5th* assessment report (RCP4.5 being most similar to the B1 scenario) suggest that linear changes in temperature and growing season length are likely (Meehl et al. 2007; Collins et al. 2013). A linear increase in mean temperature is expected under these scenarios until approximately 2060, when the rate of change slows (Meehl et al. 2007). We calculated the linear change in WSIswe and growing season length in each grid cell in the study region from 1961 to 2002 and 1950 to 1999, respectively, and extrapolated this using the cell‐specific model equations to predict changes in these variables in each grid cell (10 × 1 km) up to 2049. The mean WSIswe from 1961 to the beginning of the decade of interest and the mean growing season length from 1950 to the beginning of the decade of interest were included in models for forecasted predictions. The land cover and land use footprint for the 2000s decade were maintained in future projections. We compared future distributions to the predicted distribution calculated for the 2000s decade, using prevalence in the training data set to convert model probabilities to presence or absence (Liu et al. 2005).

## Results

### Hindcasting

The range of climate variables and the proportion of deciduous forest were similar for the validation data sets and the training data (Table 1). The validation data sets, however, contained higher total footprint and higher proportions of wetland land cover than the training data (Table 1). The missing year for harvest for 30% of the cut‐block data had little effect on the interpretation of predictions. Adding the undated cut blocks in any decade only changed 1% of the grid cells from below 0.73 to above 0.73, which was the threshold for identifying a presence, according to the prevalence in the data (Fig. 4). For this reason, we included the maximum cut‐block footprint in calculations of predictions for all tests.

**Table 1:** Value ranges for model covariates

Variable description	Training data			Aerial surveys 2000s			Aerial surveys 1990s			Aerial surveys 1980s			Aerial surveys 1970s		
	Min	Max	Mean	Min	Max	Mean	Min	Max	Mean	Min	Max	Mean	Min	Max	Mean
Growing season (days)	142.60	178.10	163.70	148.40	173.40	164.50	150.50	170.90	165.00	153.70	172.30	165.60	157.80	168.90	163.30
Winter severity	66.87	193.94	119.26	78.80	180.20	117.30	84.40	172.80	112.50	74.70	170.30	113.10	87.90	129.60	108.20
Total footprint (proportion)	0.00	0.91	0.12	0.00	1.00	0.32	0.00	1.00	0.31	0.00	1.00	0.36	0.00	1.00	0.41
Deciduous (proportion)	0.00	0.90	0.24	0.00	0.60	0.23	0.00	0.62	0.16	0.00	0.74	0.20	0.00	0.74	0.23
Wetland (proportion)	0.00	0.13	0.02	0.00	0.34	0.08	0.00	0.49	0.09	0.00	0.34	0.06	0.00	0.25	0.04

Training data are from Dawe et al. (2014).

#### Model validation

There is a temporal trend in the accuracy of the SDM predictions such that the model performs better when predictions are made closer to the time period in which the model was built (Table 2). Based on AUC, the model discrimination power was very good in the 2000s, good for the 1980s and 1990s, and poor in the 1970s. According to TSS, which weighs sensitivity and specificity as equally important, the model has high predictive power for the 1990s and 2000s decades only. The loss in predictive power of the model results from prediction of presence where the validation sets recorded absence (i.e., lower specificity) (Table 2). The sensitivity, or ability of the model to correctly predict presences in the validation data, remains high and relatively stable across decades (Table 2).

**Table 2:** Accuracy statistics, including sample size (*N*), the number of presences (*N*[p]), prevalence (prev), calculated by (*N*[p]/*N*), area under the curve (AUC), the true skills statistic (TSS), and standard deviation (SD)

Data set	*N*	*N*(p)	Prev	AUC	TSS	Sensitivity	SD	Specificity	SD
Training	299	218	0.73	0.9	0.63	0.79	0.02	0.84	0.04
2000	72	51	0.71	0.90	0.58	0.96	0.03	0.62	0.12
1990	136	78	0.57	0.8	0.49	0.95	0.03	0.41	0.07
1980	354	209	0.59	0.8	0.33	0.91	0.02	0.38	0.04
1970	113	81	0.72	0.6	0.14	0.93	0.03	0.22	0.07

The calibration model had a significant positive slope (slope = 6.64, *P* < 0.001) indicating the SDM model overestimates the proportion of occupied cells at high probabilities and underestimates the proportion of occupied cells at low probabilities. There is also a significant negative intercept (intercept = −3.55, *P* = 0.001) which indicates a tendency toward overestimating occurrence over all (i.e., there are more absences than the model predicts). This is consistent with the error found in the discrimination statistics.

#### Spatially and temporally explicit change

**Figure 3:**
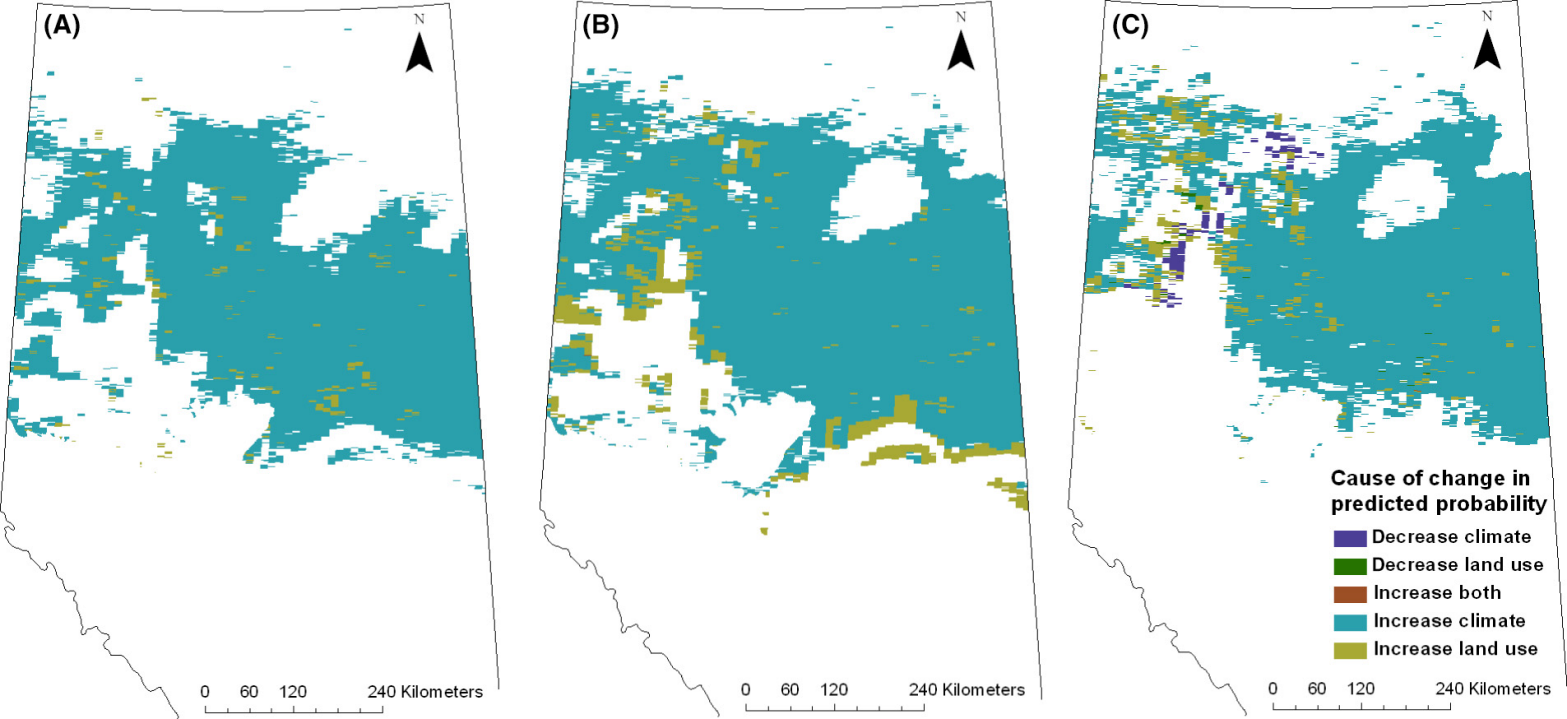
Drivers of white‐tailed deer range change between 1970 and 1980 (A), 1980 and 1990 (B), and 1990 and 2000 (C) decades. Colors indicate the variable that changed and had the largest impact on the predicted probability of white‐tailed deer presence.

The predicted probability of white‐tailed deer presence increased in 40% or more of the study area between each decade (Fig. 3). The greatest change occurred between 1980 and 1990 (54%), compared to the previous decade (42%) and the one that followed (40%). The change in predicted white‐tailed deer distribution was driven primarily by changes in climate (Fig. 3). Between 1970 and 1980, climate, rather than land use, increased probability of presence in 96% of the area that predicted an increase in white‐tailed deer presence (Fig. 3A). Similarly, the increased probability of white‐tailed deer presence in 89% and 90% of the grid cells was driven by changes in climate between 1980 and 1990 and 1990 and 2000, respectively (Fig. 3B and C). Climate was also the driver responsible for 75–80% of the cases where changes in probability of presence crossed the 0.73 threshold, which would change the interpretation from an absence to a presence (1970–1980 = 420 grid cells/527 grid cells that changed, 1980–1990 1626 grid cells/1950 grid cells, 1990–2000 = 536 grid cells/668 grid cells that changed).

**Figure 4:**
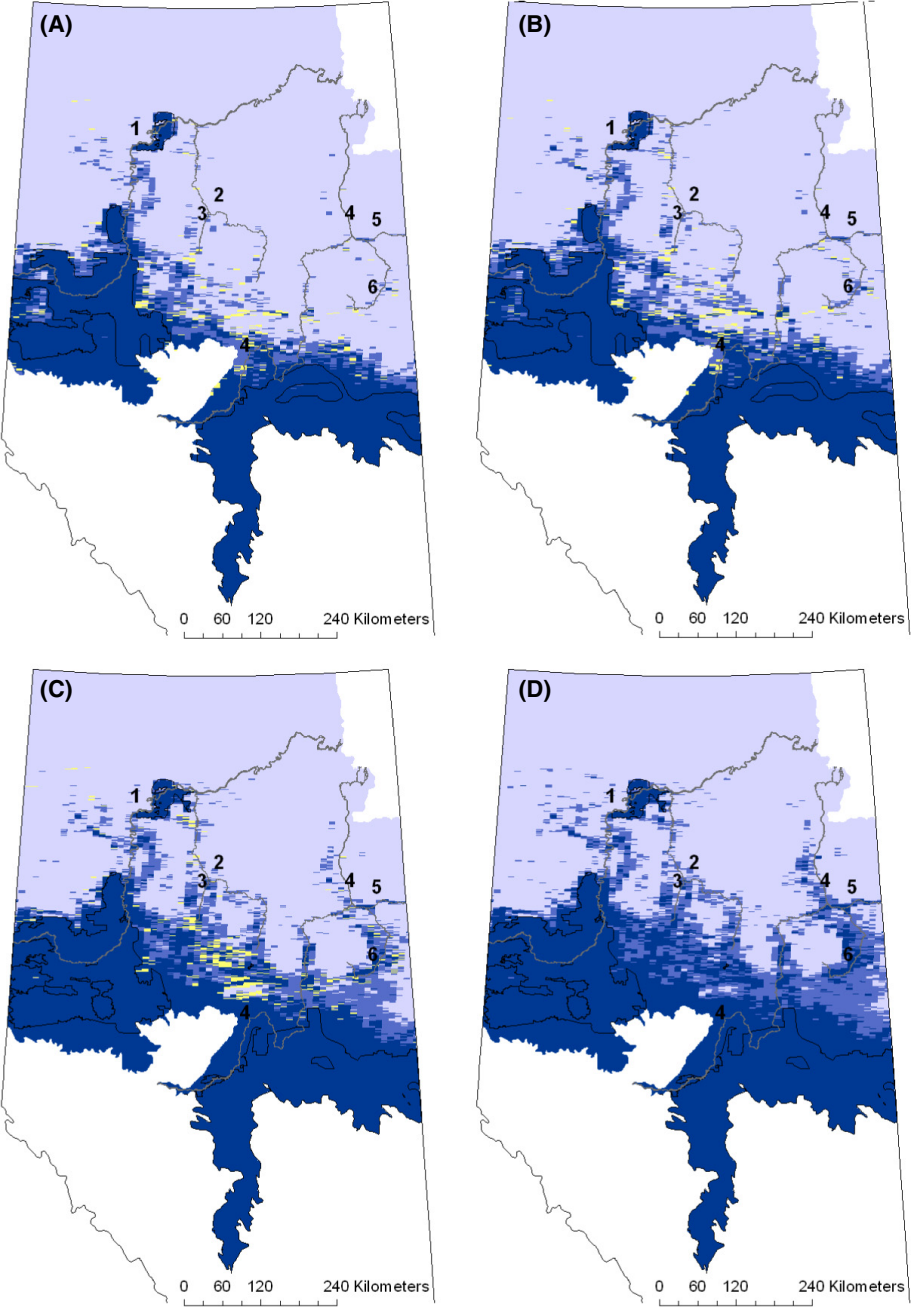
Predicted distribution of white‐tailed deer for 1970 (A), 1980 (B), 1990 (C), and 2000 (D) decades. Color indicates probability of white‐tailed deer presence is greater than or equal to 0.73 (dark blue), between 0.37 and 0.72 (medium blue), or less than 0.37 (light blue). Yellow indicates where inclusion of undated cut blocks would change the probability of presence from <0.73 to ≥0.73. The numbers correspond to rivers as listed in the caption for Figure 2. The agricultural boundary is outlined in black.

Substantially more change in predicted presence of white‐tailed deer has occurred in the northeast part of the province compared to the northwest. Based on a threshold of 0.73 to signify presence, since 1970, the distribution has moved approximately 50 to 250 km north in the eastern part of the study area (Fig. 4).

### Forecasting

Overall change in climate variables was toward warmer winters and longer growing seasons. WSIswe change ranged from a decrease of 0.155–2.248 index points per year and was greatest in the northeast portion of the study area. The largest changes in length of the growing season occurred in the northeast and far north of the study region and ranged from a shortening of 0.059 days per year to a lengthening of 0.375 days per year. Extrapolated to future decades, this resulted in a drop of 31 WSIswe index points and an increase in growing season by 5.17 days, averaged over the study area between 2000s and 2050s.

**Figure 5:**
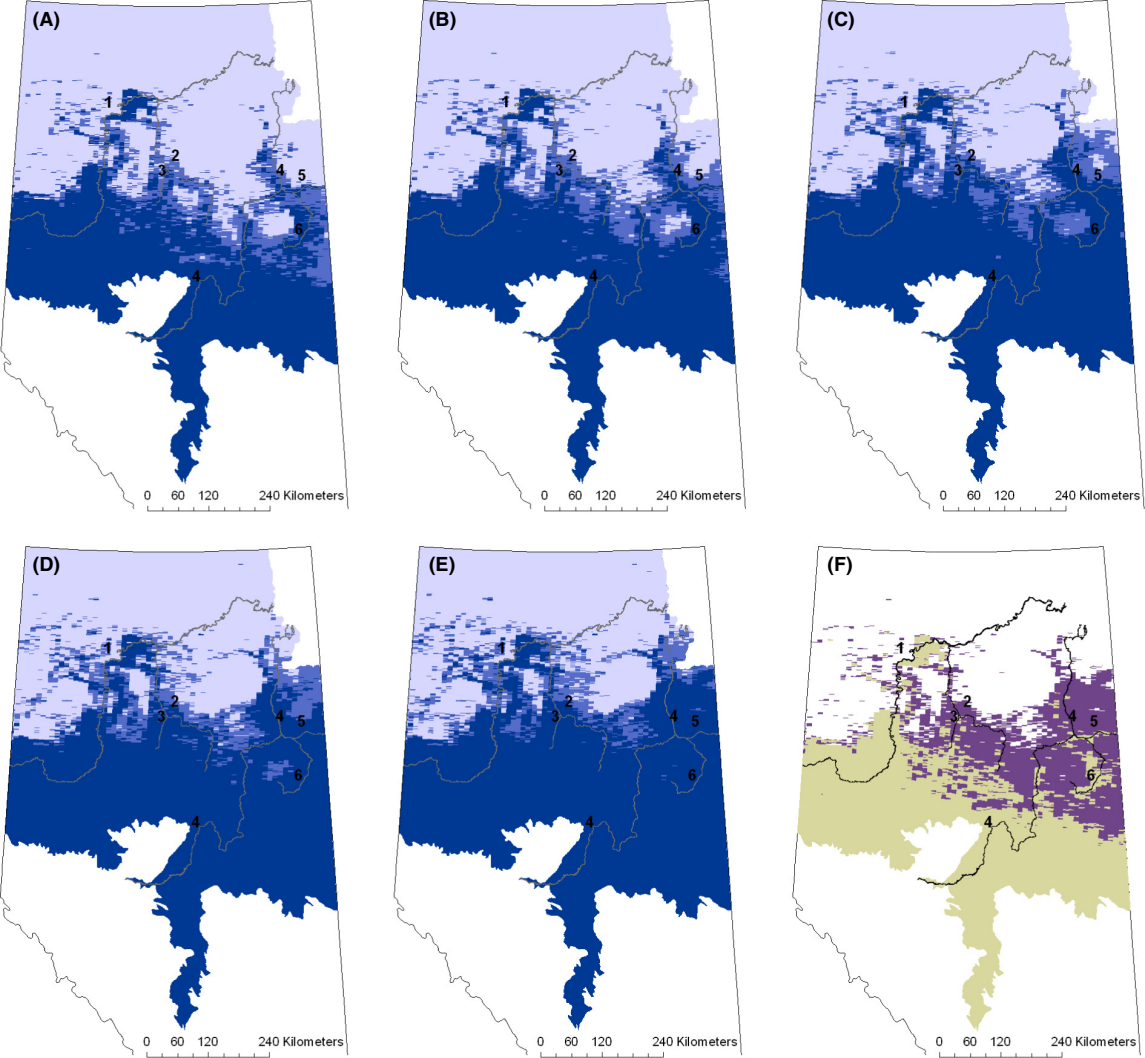
Predicted decadal distributions for 2010s (A), 2020s (B), 2030s (C), 2040s (D), and 2050s (E). Blue colors designate where predicted probability of white‐tailed deer presence is greater than or equal to 0.73 (dark blue), between 0.37 and 0.72 (medium blue), or less than 0.37 (light blue). The numbers correspond to the Peace (1), Wabasca (2), Loon (3), Athabasca (4), Clearwater (5), and Christina (6) rivers. The difference in area with a predicted probability of presence greater than or equal to 0.73 between 2050 (purple) and 2000 (beige) is shown in (F).

Predicted climate change leads to an 80,900 km^2^ increase in predicted white‐tailed deer range size in the northern Alberta boreal (Fig. 5F). Over the 2010 decade, white‐tailed deer are predicted to continue expanding along the Loon, Wabasca, Athabasca, and Christina *r*ivers, as was predicted to have occurred during the last half of the *20th* century (Fig. 5A). By the end of the 2020s, the area east of Christina River to the border of Saskatchewan is predicted to be occupied (Fig. 5B). Over the 2030s and 2040s, the distribution is predicted to extend east and west from these rivers, essentially filling in between the fingers of expansion along the major rivers in the region (Fig. 5C and D). By the end of the 2050s, white‐tailed deer are predicted to occupy most of the region south of Fort McMurray, as well as agricultural regions and major river valleys north of Fort McMurray (Fig. 5E). The quadrant formed by the southern edge of the boreal shield, the Athabasca and Christina rivers, and the Saskatchewan border is also predicted to be occupied. The distribution in the northeast is predicted to extend more than 100 km further north along the Athabasca River, while the northern extent predicted for the High Level area was predicted to have been reached during the 2000s decade. The predicted probability of white‐tailed deer presence north of High Level remained low throughout the first half of the *21st* century (Fig. 5).

## Discussion

The predicted distribution of white‐tailed deer changed substantially in northern Alberta from 1970 to 2000, particularly in the NE region of the province. Climate change, represented by decreasing severity of winter conditions and increasing length of the growing season, increased the probability of presence across substantially more of the landscape than did land use change in all decades and further changes in white‐tailed deer distribution are expected across the northern Alberta boreal forest as climate continues to change over the first half of the 21st century. Ungulates undergo periods of negative energy balance when fat stores become critical for survival (Dumont et al. 2005). It is expected that changes in climate positively affect this critical energy balance. While winter has traditionally been considered the period of energy depletion, recent work suggests individual‐based strategies for managing energy budgets, whereby females can accumulate fat in winter and deplete stores in summer to meet the energetic demands of lactation and reproduction (Monteith et al. 2013). In any case, seasonal effects on nutrition also influence survival (Monteith et al. 2013). Both winter and summer conditions contribute to the climate effect detected here. Dawe et al. (2014) found a 7.27‐fold increase in the odds of white‐tailed deer presence for a standard deviation (24.8 index units) decrease in WSIswe, while there was a 2.67‐fold increase in the odds of presence for each 5.16 days increase in the length of the growing season. Changes in winter severity and growing season length were also greatest in the northeastern region of Alberta, where changes in deer abundance and distribution have been most pronounced (Dawe 2011; Latham et al. 2011).

While climate change has been the dominant driver of expansion through much of the Alberta boreal, the positive relationship with land use footprint suggests that deer will spread into developed landscapes as the climate becomes more favorable. In areas where climate remains severe (i.e., northern extents of the study area), increasing land use intensity could also lead to white‐tailed deer spread. Northward expansion of the industrial footprint in Alberta is not currently expected (Hamley 1992; National Energy Review Board 2006); however, energy sector development in Canada's Territories (NWT, Yukon), which share the northern border with Alberta, is increasing. If the developing energy sector footprint reaches high enough intensity (>0.61 within grid cells according to Dawe et al. 2014), in the southern regions of the territories, it is plausible that it could further facilitate the expansion in those regions.

It would be expected that as climate becomes more favorable, the distribution change should also follow the pattern of natural favorable habitat. Some studies have found that white‐tailed deer disperse along river corridors, although others have not found any association (Sparrowe and Springer 1970; Long et al. 2010). The pattern predicted here indicates the probability of presence increasing northward along the Peace, Loon, Wabasca, Athabasca, and Christina rivers. The most northerly occurrence record of white‐tailed deer was also along a major river, the MacKenzie River near Fort Good Hope in Northwest Territories and two other sightings were reported further south in that river valley (Veitch 2001). The predicted distribution for the 1960s was similar to the distribution shown by Webb (1967), developed from interviews with homesteaders, surveyors, guides, trappers, and Fish and Wildlife Officers. Webb (1967) showed the distribution starting to move north along the Athabasca River in the northeast part of the province, and the provincial management plan for white‐tailed deer suggests white‐tailed deer were often sighted around Peace River, Alberta (Fig. 2; Alberta Environmental Protection 1995). These river valleys consist largely of deciduous forest, surrounded by peatlands.

The relative influence of climate change and land use on abundance may be different than that delineated here for presence. There is some expectation that the probability of detecting a species should increase as its abundance increases; however, it may be that climate change is a stronger facilitator of spread, through increased survival further northward, while land use, via provision of resources of varying quality and quantity, is a larger driver of abundance. A broad suite of factors are known to influence abundance, such as life history, competition, habitat quality, and site history, of which presence–absence models may be less influenced (Pearce and Ferrier 2001; Neilsen et al. 2005; Howard et al. 2014). Likely because of this, environmental variables have been found to be poor predictors of abundance in general (Pearce and Ferrier 2001). Neilsen et al. (2005) found that environmental variables were useful predictors for moose abundance, at some densities, however, and they suggested that when resources are patchy on the landscape, presence–absence and abundance should be more closely related. Howard et al. (2014) also found that predictive accuracy of SDMs was improved when they were trained with abundance data, likely because it provides a proxy for habitat quality, which is lost in presence–absence data.

The quality of resources for white‐tailed deer resulting from different land use footprints is likely patchy across the study area. Land use footprint was included in models as the cumulative total footprint; however, the individual industry contributions varied substantially over space and time (Dawe et al. 2014). The agricultural industry developed the largest proportion of the boreal forest and the quality of food obtained from crops, such as barley, canola, wheat, and oats, is higher than that available in the traditional boreal forest ecosystem (Hamley 1992). Agriculture is comparatively absent from the eastern half of the Alberta boreal forest, where abundance remained low until recent years (Latham et al. 2011). This leads to the suggestion that land use patches may serve as a source for spread into surrounding landscapes. Dawe et al. (2014) did not find evidence that distance from agriculture was important in describing presence; however, this may be important for predicting abundance over time and space. It is important to investigate the relative influence of factors driving presence and abundance separately because management implications for presence differ from those for abundance. Current management approaches may not allow us to manage white‐tailed deer spread, given the large influence of changing climate; however, traditional management methods can be applied to manage local abundance, which may mitigate some of the negative effects to native species. There have been calls to improve our collection of and modeling approaches with abundance data and a number of recent advances have been made which may allow for incorporation of indexed abundance data in future informative analyses (Howard et al. 2014).

The SDM predictions for past decades matched closely with the observed presences and absences from independent validation data; however, predictive power decreased as predictions were made further from the decade in which the model was built. This loss of predictive power appears to be related to the model's ability to predict absences. In 1970, only 38% of absences in validation data were predicted to be absences. This means 62% of absences in the data were predicted to have white‐tailed deer present. Araújo et al. (2005) suggested temporal autocorrelation in predictions is reduced the further apart in time the predictions are made, which could lead to decreased predictive power across time. However, tests of accuracy also rely on the validation data consisting of perfect species detection, which is rarely possible with ecological data (Lobo et al. 2008). Canopy cover, species activity, population size, and observer bias, among other factors, can contribute to imperfect detection during field surveys (Royle et al. 2005). Absences in the validation data resulting from nondetection would lower the apparent specificity of the model predictions. Further, lag times in species' responses to climate changes, interactions with predators or competitors, or seasonal fluctuations in abundance, survival, and reproduction could also lead to actual species absences from otherwise suitable locations (Araújo et al. 2005; Mitikka et al. 2008). If training data include imperfect absences due to detection error, the model may predict absences where there are actually species present (false negatives), leading to decreased sensitivity in the model (Lobo et al. 2008). The data were collected using snow‐tracking methods that integrate species presence over the 3–10 day period after snowfall (Dawe et al. 2014). This likely minimizes recording of false absences at all but very low abundances. Sensitivity remained high through all decades tested, which suggests this was not a major concern for the predictive performance of the SDM. Accuracy in prediction of absences is less important for some applications of SDMs than for others (Araújo et al. 2005; Allouche et al. 2006; Lobo et al. 2008). White‐tailed deer are already having negative impacts in northern Alberta (Latham et al. 2011). The consequences of the white‐tailed deer SDM overpredicting the absolute probabilities of occurrence (calibration error) by underpredicting absences (low specificity in past predictions) are arguably lower than the consequences of expecting them to be absent when they are present.

A trade‐off exists when including mechanistically informed climate variables in SDMs because the variables may not be included in GCM projections under future climate scenarios. Although projections from GCMs are based on varying parameterizations and complexity of atmospheric and ocean circulation systems, several authors have modeled change in climate variables over past decades using linear relationships (Stone et al. 2002; Rikiishi et al. 2004; Mote et al. 2005; Randall et al. 2007; Ashcroft et al. 2009; Heffernan 2010). Rates of change averaged over recent decades closely match those predicted for the near future. Although there is variability when averaged over shorter time periods, temperature increases of 0.11°C/decade were observed between 1951 and 2012. Temperature projections under all representative concentration pathways assessed in the 2014 assessment report are similar for the near‐term period (2016–2035), with temperature increases projected to be between 0.12 and 0.43°C/decade (Kirtman et al. 2013). After the 2030s, predictions under different emissions scenarios begin to diverge, according to multimodel averages (Meehl et al. 2007). The future projections presented here are based on an assumption that climate parameters continue to change in the same direction and magnitude as they have, on average, since the 1950s. Given the strong predictive ability of the SDM in decades close to the training data collection period, and consistent projections from climate models into near‐term periods, we expect that predictions through the 2030s to be sound. Decline in SDM reliability over longer timescales and the importance of emissions scenarios and representative concentration pathways on longer term climate change projections suggest the linear modeling approach taken here may lead to more uncertain predictions for the 2040s and 2050s. Near‐term predictions are helpful for policy and management decision making, however, while the longer term predictions provide a possible outcome if white‐tailed deer management is not considered. Explicitly defining locations where climate change is the driving factor for species distribution change allows adaptation plans to be developed to manage the changing mammal community. Defining areas where climate remains severe and land use development could lead to further deer distribution change allows decisions to be made to manage and reclaim new and existing footprint to minimize the expansion. By spatially and temporally delineating the changes driven by the competing mechanisms of climate change and land use, we quantify the relative influence of two dominant factors affecting current and future species distributions. This information is critical for incorporating climate change into management and adaptation plans and anticipating further changes across the landscape.

## Conflict of Interest

None declared.
